# Prediction of neoadjuvant chemotherapeutic efficacy in patients with locally advanced gastric cancer by serum IgG glycomics profiling

**DOI:** 10.1186/s12014-020-9267-8

**Published:** 2020-02-06

**Authors:** Ruihuan Qin, Yupeng Yang, Hao Chen, Wenjun Qin, Jing Han, Yong Gu, Yiqing Pan, Xi Cheng, Junjie Zhao, Xuefei Wang, Shifang Ren, Yihong Sun, Jianxin Gu

**Affiliations:** 1 NHC Key Laboratory of Glycoconjugates Research, Department of Biochemistry and Molecular Biology, School of Basic Medical Sciences, Fudan University, Shanghai, 200032 China; 2 Chinese Institute for Brain Research, Beijing, 102206 China; 3 Department of General Surgery, Zhongshan Hospital, Fudan University, Shanghai, 200032 China; 4 Department of Medical Oncology, Zhongshan Hospital, Fudan University, Shanghai, 200032 China

**Keywords:** Gastric cancer, Neoadjuvant chemotherapy, IgG glycosylation, Efficacy prediction, UPLC

## Abstract

**Background:**

Neoadjuvant chemotherapy (NACT) could improve prognosis and survival quality of patients with local advanced gastric cancer (LAGC) by providing an opportunity of radical operation for them. However, no effective method could predict the efficacy of NACT before surgery to avoid the potential toxicity, time-consuming and economic burden of ineffective chemotherapy. Some research has been investigated about the correlation between serum IgG glycosylation and gastric cancer, but the question of whether IgG glycome can reflect the tumor response to NACT is still unanswered.

**Method:**

Serum IgG glycome profiles were analyzed by Ultra Performance Liquid Chromatography in a cohort comprised of 49 LAGC patients of which 25 were categorized as belonging to the NACT response group and 24 patients were assigned to the non-response group. A logistic regression model was constructed to predict the response rate incorporating clinical features and differential *N*-glycans, while the precision of model was assessed by receiver operating characteristic (ROC) analysis.

**Results:**

IgG *N*-glycome analysis in pretreatment serum of LAGC patients comprises 24 directly detected glycans and 17 summarized traits. Compared with IgG glycans of non-response group, agalactosylated *N*-glycans increased while monosialylated *N*-glycans and digalactosylated *N*-glycans decreased in the response group. We constructed a model combining patients’ age, histology, chemotherapy regimen, GP4(H3N4F1), GP6(H3N5F1), and GP18(H5N4F1S1), and ROC analysis showed this model has an accurate prediction of NACT response (AUC = 0.840) with the sensitivity of 64.00% and the specificity of 100%.

**Conclusion:**

We here firstly present the profiling of IgG *N*-glycans in pretreatment serum of LAGC. The alterations in IgG *N*-glycome may be personalized biomarkers to predict the response to NACT in LAGC and help to illustrate the relationship between immunity and effect of NACT.

## Introduction

Gastric cancer is one of the most aggressive gastrointestinal malignancy, and third leading cause of cancer deaths worldwide due to a frequent diagnosis at advanced stages which remain to be a non-curative state [1]. Fortunately, neoadjuvant chemotherapy (NACT) provides an opportunity of radical operation for patients with local advanced gastric cancer (LAGC). NACT for gastric cancer can reduce the size of tumors, down-stage tumors, and reduce the tumor-related symptoms, thereby increasing curative resection rate and improving survival rate [2, 3]. However, the overall response rate to chemotherapy is less than 50% and non-effective chemotherapy would bring side effect such as toxicity, wasting of time and money [2]. If these patients are not benefiting from preoperative treatment, alternative therapies may be offered at an earlier stage [4]. Thus, in order to improve the quality of life of non-responders, avoid potential toxicity, reduce the time until surgery and reduce cost, it is necessary to find a biomarker to predict the efficacy of NACT before treatment.

Recently, several studies have reported that the immune response plays an important role in the patients with LAGC who received NACT. Some immunologic markers were used to evaluate the response of NACT. LAGC patients with low SII (neutrophil × platelet/lymphocyte), low NLR (neutrophil/lymphocyte ratio) or low PLR (platelet/lymphocyte ratio) in pre-treatment serum seems have better NACT efficacy [5–7]. In addition, He et al. studied the impact of the immune cell population in peripheral blood and found high CD3+ CD8+ T cells, and low CD4+ CD25+ Foxp3+ Tregs could be biomarkers to identify patients likely to benefit from NACT [8]. Although great efforts have been made to identify markers whose expression is associated with tumor response to chemotherapy, no markers with sufficient sensitivity and specificity have been developed for a clinical application so far.

Immunoglobulin G (IgG), the most abundant glycoprotein in the serum, is the key molecule in humoral immunity of many diseases [9]. The effector functions of IgG were influenced by *N*-glycosylation at the conserved site of the Fc fragment [10, 11]. Differential glycosylation such as fucosylation, sialylation, and galactosylation was discovered in both total serum IgG and disease-specific IgG in gastric cancer [12–14]. Aberrant IgG glycosylation could be potential biomarkers in early detection and progress surveillance of gastric cancer [15, 16]. However, the less is known about the potential role of the IgG glycosylation in tumor immunity and NACT efficacy.

Accordingly, the aims of this study are to analyze the relationship between IgG glycosylation and the response to NACT in patients with LAGC and evaluate the possible value of IgG glycosylation in neoadjuvant chemotherapeutic efficacy prediction. In this study, we conducted the analysis of various of IgG glycan expression in response group and non-response group to NACT and built a logistic regression model combining altered IgG glycosylation with clinical characteristics to predict the response rate of NACT in patients with LAGC.

## Materials and methods

### Study population and sample collection

Two hundred and forty-six serum samples from advanced gastric cancer patients were collected from Department of General Surgery, Zhongshan Hospital, Fudan University, Shanghai, China, from January 2016 to January 2017. All patients had given informed consent and met the eligibility criteria: (1) 18 years < Age < 80 years; (2) Patients were diagnosed with LAGC; (3) Patients meet the clinical condition for NACT and planned gastrectomy; (4) All clinical characteristics and pathological information were available, such as NACT relevant information, imaging information, and pathological diagnosis. The exclusion criteria were: (1) patients who ever received chemoradiotherapy; (2) patients who ever received gastrectomy; (3) patients who had simultaneously developed other tumor; (4) image examination had been performed to assess clinical stage with all patients having cT2-4 and (cN+ or cN−), according to Habermann et al.’s method [17]. Finally, only 49 patients who underwent NACT were included among 246 gastric cancer patients. Human blood serums were collected from these gastric cancer patients before the first cycle of preoperative chemotherapy. All serum layer was collected and stored at − 80 °C until analysis. No more than three cycles of freezing/thaw were allowed for any sample. Clinical data from the patients are summarized in Table 1. Approvals were obtained from the Institutional Review Board and informed written consents from all participants were acquired.

**Table 1:** Clinicopathological characteristics of all patients

Characteristics	Total (%)	Non-response group (%)	Response group (%)	P value
Age				0.884
≤ 60	25 (51)	13 (54)	12 (48)	
> 60	24 (49)	11 (46)	13 (52)	
Sex				0.924
Male	34 (69)	16 (67)	18 (72)	
Female	15 (31)	8 (33)	7 (28)	
Site				0.291
U	11 (22)	6 (25)	5 (20)	
M	15 (31)	8 (33)	7 (28)	
L	16 (33)	9 (38)	7 (28)	
Overlapping	7 (14)	1 (4)	6 (24)	
Histology				0.479
Adenocarcinoma	11 (22)	4 (17)	7 (28)	
SRC	6 (12)	4 (17)	2 (8)	
Other	32 (65)	16 (67)	16 (64)	
Regimen				0.686
DOS	10 (20)	6 (25)	4 (16)	
XELOX	23 (47)	11 (46)	12 (48)	
Other	16 (33)	5 (21)	8 (32)	

### Neoadjuvant chemotherapy

All patients received 2 to 5 cycles of chemotherapy, in which 10 patients suffering docetaxel oxaliplatin and S-1 (DOS) regimen, 23 patients suffering capecitabine and oxaliplatin (XELOX) regimen and 16 patients suffering other regimens.

### Evaluation of histopathological response

The histopathological response was evaluated by the Becker regression score according to the estimation of the percentage of residual tumor tissue, including four grades: 1a, complete tumor regression; 1b, less than 10% vital tumor; 2, 10–50% residual tumor; and 3, more than 50% remaining residual tumor [18]. All patients with Grade 1a, Grade 1b, or Grade 2 regression were regarded as response group, while Grade 3 was classified as non-response group.

### IgG glycans measurement

#### IgG purification from human plasma

Purification of IgG was described in the previous study [19, 20]. IgG from serum sample was isolated using Protein A IgG Purification Kit (Thermo Fisher Scientific, Rockford.). The isolation was manipulated according to the manufacturer instructions. Briefly, 50 μL of serum was diluted 2× with IgG Binding Buffer and applied to the protein A plate. IgG was eluted with 400 μL IgG Elution Buffer and neutralized with 40 μL IgG Binding Buffer. And the fractions containing IgG were stored at − 20 °C until the *N*-glycans release.

#### IgG *N*-glycans release, enrichment and labelling

As described in the previous study, IgG *N*-glycans were released from IgG-containing elution by incubating with 1 μL PNGase F (New England Biolabs, Inc.) for 12 h at 37 °C [19]. Subsequently, the released *N*-glycans were purified by porous graphic carbon (PGC) solid-phase extraction. Briefly, a PGC-containing 96-well plate was washed with 200 μL of 0.1% trifluoroacetic acid (TFA) (v/v) in 80% acetonitrile (ACN) (v/v) and followed by 0.1% TFA (v/v). The solution of released *N*-glycans was applied to the PGC-containing 96-well plate three times to allow complete *N*-glycans adsorption. Then, H_2_O was added to remove salts and buffer. The *N*-glycans derived from IgG were eluted with 100 μL of 0.05% TFA (v/v) in 25% ACN (v/v).

Then the elute was dried in a concentrator (Eppendorf) for 3 h on 45° manual mode followed by labeling with 2-aminobenzamide (2-AB) described by Maja Pucic et al. [21]. The labeling mixture was freshly prepared by dissolving 50 mg 2-AB (Sigma-Aldrich) and 60 mg Sodium cyanoborohydride (Sigma-Aldrich) in 1 mL of 0.7% DMSO (Sigma-Aldrich) and 0.3% glacial acetic acid (Merck) (v/v). A volume of 3 μL of labeling mixture was added to each *N*-glycans sample. Mixing was achieved by shaking for 2 min, followed by 2 h incubation at 60 °C. The labeling reaction was stopped by adding 50 μL H_2_O per sample.

#### Hydrophilic interaction chromatography (HILIC)-UPLC

The labeled *N*-glycans were separated by HILIC on a Nexera UHPLC LC-30A (Shimadzu) with fluorescence detector set with excitation and emission wavelengths of 330 and 420 nm, respectively. The instrument was under the control of LabSolution software (Shimadzu). Labelled *N*-glycans were separated on a Waters BEH Amide chromatography column (Waters), 100 × 2.1 mm i.d., 1.7 μm BEH particles, with 100 mM ammonium formate, pH 4.5, as solvent A and ACN as solvent B. Separation method used linear gradient of 79–56% ACN (v/v) at a rate of 0.5 mL/min in a 26 min analytical run. Samples were maintained at 4 °C before injection, and the separation temperature was 70 °C. Data processing was performed using an automatic processing method with a traditional integration algorithm after which each chromatogram was manually corrected to maintain the same intervals of integration for all the samples. The chromatograms were all separated in the same manner into 24 peaks. Relative intensities of each glycan structures in each UPLC peak were determined by mass spectrometry in previous literatures [21, 22]. According to the same structural features such as fucosylation, galactosylation, sialylation and bisecting type of *N*-glycosylation from related studies, 17 summarized traits were calculated that average these features across multiple glycans [23, 24] (Additional file 1: Table S1).

### Data normalization and statistical analysis

In order to normalize the measurement of glycans, each peak area of glycan was divided by total area of the corresponding chromatogram. Chi-squared test and Fisher’s exact test were used for binary and categorical variables, as appropriate. T-test was chosen for continuous variables. Patients clinical characteristics were compared between the response group and non-response group. A multivariate logistic regression was conducted to predict the neoadjuvant chemotherapy response and stepwise regression was used to perform variable selection. However, critical clinical variable, such as age, histology and chemotherapy regimen, were also included in multivariate analysis considering the clinical significance. Receiver operating characteristic curve (ROC) analysis was used to investigate the diagnostic value of the model. All the statistical analyses were performed using R 3.4.3 software and IBM SPSS Statistics version 20.0 software. In all test, two-sided P values smaller than 0.05 were considered statistically significant.

## Results

### Patient characteristics and NACT response

The descriptive information of 49 patients with LAGC was presented in Table 1. Mean age before NACT was 57.59 years and 69% were male. All patients were clinical TNM stage III and underwent surgery after NACT. All patients were assessable for pathologic response. Tumor regression was grade 1b in 10 patients (20.41%), grade 2 in 15 patients (30.61%), and grade 3 in 24 patients (48.98%). Therefore, 25 (51.02%) patients were categorized as pathologic responders (response group) and 24 (48.98%) categorized as pathologic non-responders (non-response group).

Correlation analysis showed that there was no relationship between patients’ age, sex, tumor site, histology and chemotherapy regimen and the response rate after NACT, which suggested that clinical characteristics may not enough to predict the efficacy of NACT along.

### Serum IgG *N*-glycome in patients with LAGC before preoperative chemotherapy

**Fig. 1:**
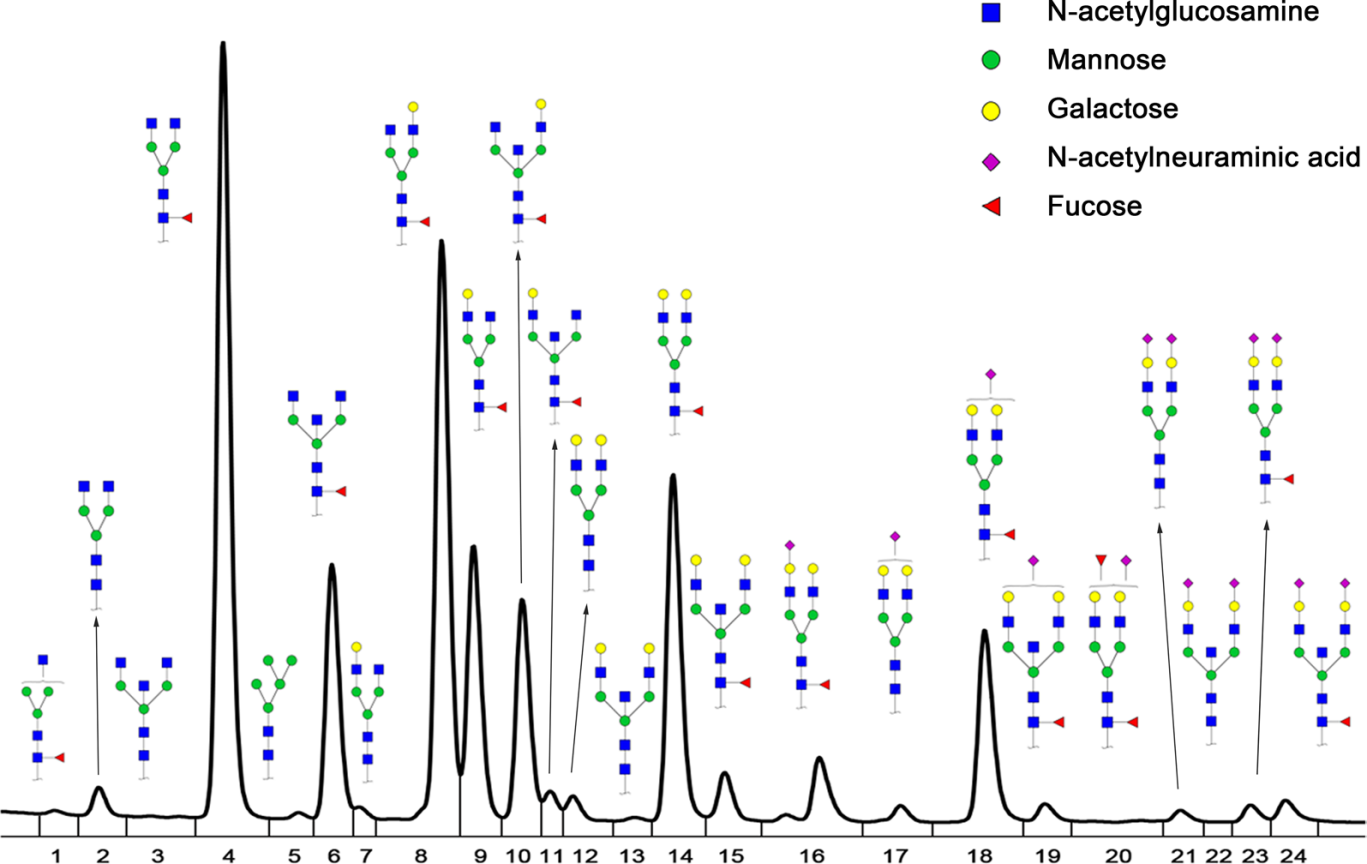
Representative Ultra Performance Liquid Chromatography (UPLC) chromatogram of serum IgG *N*-glycan profiles. A total of 24 chromatographic peaks were shown

IgG glycome composition was detected by UPLC analysis of 2-AB labeled glycans as described in previous literature [21]. The typical glycomics profile with 24 directly detected peaks was shown in Fig. 1, which was a representative chromatogram for serum IgG *N*-glycans of patients with LAGC before treatment. Besides the directly detected glycans, 17 traits were calculated based on the same structural features of 24 directly detected glycans (fucosylation, galactosylation, sialylation, and bisecting type *N*-glycosylation) (Additional file 1: Table S1).

**Fig. 2:**
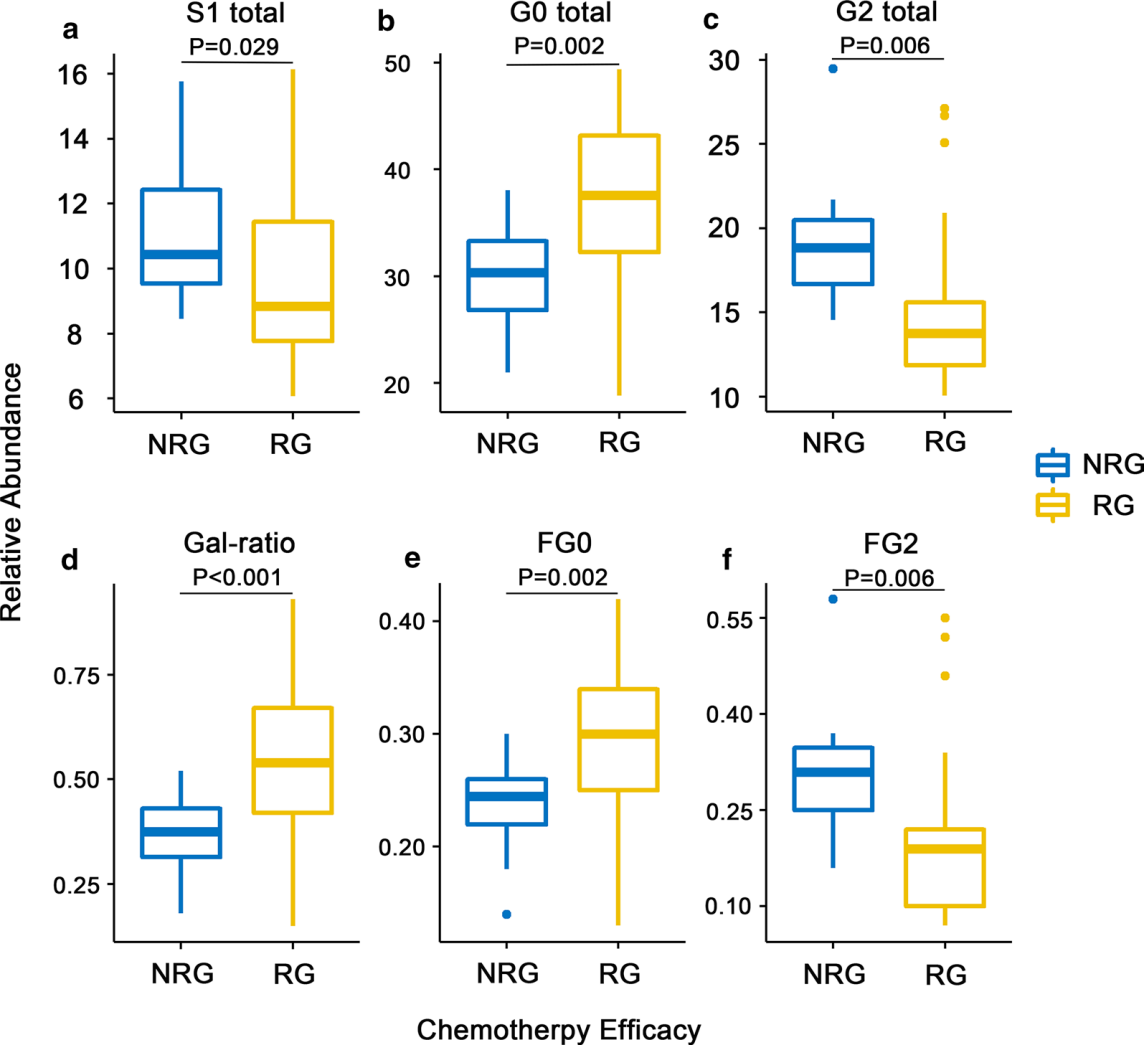
The abundance of significantly different summarized-traits in patients with response and without response to NACT. RG represents NACT response group, while NRG represents NACT non-response group. The *N*-glycans were grouped according to their structural features, **a** the proportion of sialylation in total IgG glycans (S1 total); **b** the proportion of agalactosylated *N*-glycosylation in total IgG glycans (G0 total); **c** The proportion of digalactosylated *N*-glycosylation in total IgG glycans (G2 total); **d** the ratio of agalactosylated *N*-glycosylation to galactosylated structures of fucosylated glycans (Gal-ratio); **e** the proportion of agalactosylated structures in total fucosylated glycans (FG0); **f** the proportion of digalactosylated structures in total fucosylated glycans (FG2)

In this study, six directly measured glycans were different between non-response group and response group (P < 0.05) (Table 2). Among them, GP1 (H3N3F1), GP4 (H3N4F1) and GP6 (H3N5F1) were increased, while GP14 (H5N4F1), GP15 (H5N5F1) and GP18 (H5N4F1S1) decreased in the response group. For summarized traits, neutral glycans and agalactosylated *N*-glycosylation increased while sialylation and galactosylation decreased in response group, and the fucosylation and bisecting glycosylation showed a slight fluctuation between the two groups (Table 2). Specifically, monosialylated structures (S1 total, P = 0.029) (Fig. 2a) showed a significant decreased level in the response group. Both total agalactosylated *N*-glycosylation (G0 total, P = 0.002) (Fig. 2b) and fucosylated agalactosylated *N*-glycosylation (FG0, P = 0.002) (Fig. 2e) were increased in patients who would response NACT, while total digalactosylated *N*-glycosylation (G2 total, P = 0.006) (Fig. 2c) and fucosylated digalactosylated *N*-glycosylation (FG2, P = 0.006) (Fig. 2f) were reduced. That seems to reveal the difference of total galactosylation is mainly based on the fucosylated structures. Furthermore, the “Gal-ratio” proposed in our previous study [25], represented the level of fucosylated galactosylation of IgG was significantly increased in PC group (P < 0.001)(Fig. 2d) and could be a moderately accurate marker for prediction of NACT efficacy (AUC (under area of ROC) = 0.776, 95% CI 0.633 to 0.919). Besides, the other eight altered traits also have potential to predict NACT efficacy by ROC analysis (AUC > 0.700, Table 2).

**Table 2:** IgG glycome composition in gastric cancer patients with or without response to NACT

IgG glycome	Traits (composition)	Mean NRG^a^)	Mean (RG^a^)	P value	AUC	Confidence interval (95%)
Directly detected glycans	GP1 (H3N3F1)	0.137	0.182	*0.048*	0.678	0.524–0.832
	GP2 (H3N4)	0.623	0.828	0.075	0.658	0.501–0.816
	GP3 (H3N5)	0.013	0.005	0.179	0.568	0.406–0.730
	GP4 (H3N4F1)	22.580	27.448	*0.003*	0.771	0.628–0.913
	GP5 (H5N2)	0.157	0.144	0.460	0.474	0.309–0.639
	GP6 (H3N5F1)	6.588	7.577	*0.042*	0.654	0.498–0.811
	GP7 (H4N4)	0.353	0.348	0.902	0.519	0.352–0.687
	GP8 (H4N4F1(6))	20.344	19.628	0.264	0.622	0.462–0.783
	GP9 (H4N4F1(3))	10.108	9.925	0.678	0.541	0.372–0.709
	GP10 (H4N5F1(6))	6.424	6.097	0.446	0.462	0.296–0.629
	GP11 (H4N5F1(3))	0.691	0.725	0.542	0.451	0.286–0.616
	GP12 (H5N4)	1.005	0.820	0.276	0.539	0.374–0.705
	GP13 (H5N5)	0.033	0.014	0.209	0.568	0.406–0.731
	GP14 (H5N4F1)	15.783	12.944	*0.007*	0.790	0.650–0.930
	GP15 (H5N5F1)	1.840	1.496	*0.017*	0.712	0.559–0.865
	GP16 (H4N4F1S1(3))	2.313	2.255	0.657	0.568	0.404–0.733
	GP17 (H5N4S1)	0.620	0.521	0.146	0.577	0.413–0.741
	GP18 (H5N4F1S1)	7.218	5.976	*0.028*	0.710	0.559–0.861
	GP19 (H5N5F1S1)	0.778	0.737	0.446	0.589	0.424–0.755
	GP20 (H5N4F2S1)	0.027	0.011	0.168	0.571	0.409–0.733
	GP21 (H5N4S2)	0.495	0.519	0.692	0.528	0.361–0.696
	GP22 (H5N5S2)	0.003	0.002	0.622	0.555	0.392–0.718
	GP23 (H5N4F1S2)	0.835	0.792	0.689	0.582	0.414–0.750
	GP24 (H5N5F1S2)	1.033	1.006	0.859	0.484	0.317–0.651
Main summarized traits	GPN	86.682	88.182	0.059	0.642	0.482–0.802
	S1 total	10.929	9.489	*0.029*	0.695	0.543–0.847
	S2 total	2.364	2.318	0.871	0.528	0.360–0.695
	S total	13.292	11.808	0.061	0.642	0.482–0.802
	G0 total	29.941	36.040	*0.002*	0.773	0.630–0.917
	G1 total	37.920	36.724	0.064	0.685	0.531–0.839
	G2 total	18.663	15.273	*0.006*	0.798	0.658–0.939
	F total	94.572	94.798	0.505	0.487	0.319–0.654
	F neutral	97.473	97.556	0.797	0.534	0.365–0.703
	F sialo	91.669	90.941	0.185	0.623	0.463–0.783
	B total	17.402	17.660	0.770	0.531	0.365–0.697
	B neutral	17.998	18.007	0.993	0.510	0.343–0.677
	B sialo	13.650	14.661	0.413	0.565	0.400–0.730
	Gal-ratio	0.370	0.526	*< 0.001*	0.776	0.633–0.919
	FG0	0.238	0.290	*0.002*	0.768	0.625–0.911
	FG1	0.322	0.311	0.121	0.633	0.477–0.790
	FG2	0.168	0.136	*0.006*	0.786	0.647–0.925

^a^RG, patients with response to NACT; NRG, patients without response to NACT; H, Mannose; *N*, *N*-acetylglucosamine; F, Fucose; S, *N*-acetylneuraminic acid; italic font indicates the trait exist significant difference

### Predictive model of response to NACT

**Fig. 3:**
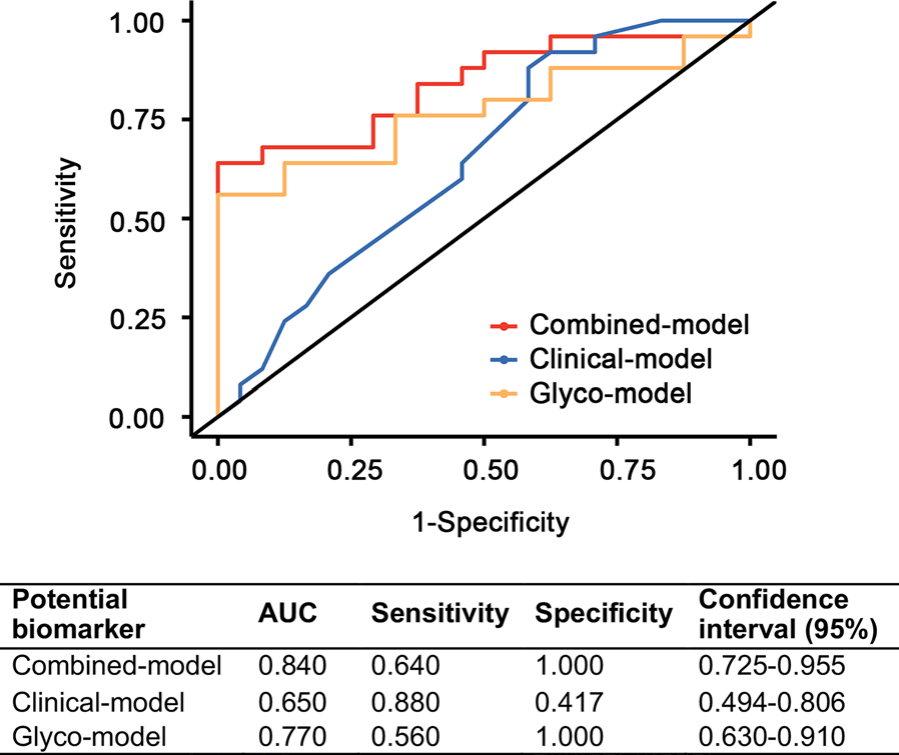
ROC curve analyses for the prediction of NACT efficacy in patients with gastric cancer. Three models were constructed to predict the NACT efficacy. The blue line represents “Clinical-model” that consisted with age, histology and regimen. The yellow line represents “Glyco-model” consisted with GP4(H3N4F1), GP6(H3N5F1), and GP18(H5N4F1S1). The red line represents “Combined-model” that combined two models variate mentioned above

In order to accurately predict the efficacy of NACT in gastric cancer, we attempted to build a predictive model including clinical traits and the directly measured IgG glycans. First, we built a clinical-model with age, histology, and regimen which usually considered could influence the chemotherapeutic efficacy. But this model could not effectively predict the outcome of NACT (AUC = 0.650, 95% CI 0.494 to 0.806). Subsequently, a glyco-model was constructed with directly detected glycans [GP4(H3N4F1), GP6(H3N5F1), and GP18(H5N4F1S1)], which presented a moderately accurate prediction of NACT response (AUC = 0.770, 95% CI 0.630 to 0.910). Finally, the combined-model was built combining glycan variables and clinical traits, and this combined model displayed a more potential clinical utility to predict responders of NACT (AUC = 0.840, 95% CI 0.725 to 0.955) (Fig. 3).

It has been reported that there is no significant difference in IgG glycome after surgical treatment [23]. In order to explore the relationship between IgG glycome and NACT, we analyzed IgG glycome composition in 40 patients before NACT and after NACT. Both in the non-response group (n = 18) and response groups (n = 22), we did not observe any significant changes in IgG glycans that were caused by the NACT (Additional file 1: Tables S2, S3).

## Discussion

This study firstly investigated the association between the IgG glycosylation in patients with LAGC who received NACT and their clinical outcome (response or non-response). Through using IgG *N*-glycomics analysis in pretreatment serum, we found neutral glycosylation and agalactosylated *N*-glycosylation increased while sialylation and galactosylation decreased in response group which is a benefit to illuminate the relationship between immunity and NACT. The combined-model including differential glycans with clinical features was promoted in our study which presents an accurate predictive performance of NACT efficacy with AUC value of 0.840 (95% CI 0.725 to 0.955). And this diagnostic model could help surgeons choose the most appropriate treatment for patients with LAGC.

In our study, clinical features including age, sex, site of tumor, histology, and regimen were compared between the response group and non-response group. Although none of these features showed significant differences, we still constructed the clinical-model included age, histology and regimen due to the clinical significance of LAGC with NACT. Noteworthy, consistent with other published studies [26, 27], less than 50% of patients showed a histologic response to NACT in our cohort, thus find a biomarker to predict the efficacy of NACT before treatment to avoid the potential toxicity, time-consuming and economic burden of ineffective chemotherapy is necessary.

As an effective biomarker screening tool, IgG *N*-glycomics analysis has been used in the diagnosis of autoimmune diseases and multiple cancers [15, 23, 28, 29]. The most significant cancer-associated changes in glycosylation are fucosylation, galactosylation, and sialylation. Depending on the extent of those glycosylation, IgG will active complement, activate antibody-dependent cellular cytotoxicity (ADCC), or even an anti-inflammatory action [30]. In gastric cancer, differential glycans such as fucosylation, sialylation, and galactosylation were discovered in both total serum IgG and disease-specific IgG [12–14]. Agalactosylated *N*-glycosylation can increase the binding with mannose-binding lectin, resulting in promotion of complement-dependent cytotoxicity (CDC) activity, while galactosylation can promote the association between IgG and Fcγ inhibitory receptors, resulting in an increase anti-inflammatory activity [13]. Besides, both monosialylated glycan and disialylated glycan of IgG play an essential role in anti-inflammatory activity in the innate immune system [31]. These differential glycans of IgG were promoted to be potential biomarkers in early diagnosis of gastric cancer and differential diagnosis of benign and malignant gastric cancer.

Our results indicated that the anti-inflammatory role of the galactosylation and sialylation of IgG might have different mechanisms or different grade inflammation between non-response group and response group. Decreased sialylated glycan and galactosylated glycan in response group suggest that relative pro-inflammatory environment seems to contribute to the response to NACT in gastric cancer. Besides, no change of inflammatory state occurred after NACT both in response group and non-response group. To our knowledge, this is the first study to demonstrate that IgG *N*-glycome may be correlated with histopathological response to NACT in gastric cancer. Some of these altered glycan traits have the potential to predict efficacy of NACT. It is worth mentioning that the potential multiple cancer types marker “Gal-ratio” proposed in our previous study also has the potential to predict NACT efficacy (AUC = 0.776, 95% CI 0.633 to 0.919).

There are some limitations to our study. We did not recruit enough number of patients underwent NACT. Further validation studies in additional sample cohorts will be needed to evaluate whether the model with IgG *N*-glycans has sufficient predictive power of response to NACT. Besides, although the regimen was considered in our combined-model, the impact of the complex regimen on IgG *N*-glycans is still not fully elucidated. Furthermore, the biological mechanism for such a relationship has yet to be determined.

Taken together, we here present the first profiling of IgG *N*-glycans in pretreatment serum of LAGC. The alterations in IgG *N*-glycome may be personalized biomarkers to predict the response to NACT in LAGC. Further study should be repeated in independent cohorts with different chemotherapy regimens in the future.

## Supplementary information

**Additional file 1: Table S1.** Summarized glycan traits based on the glycan structure. **Table S2.** IgG glycome composition in gastric cancer patients with response to NACT before and after treatment. **Table S3.** IgG glycome composition in gastric cancer patients without response to NACT before and after treatment.

## Data Availability

The datasets used and/or analyzed during the current study are available from the corresponding author on reasonable request.

## Abbreviations

NACT: Neoadjuvant chemotherapy
LAGC: Local advanced gastric cancer
SII: Neutrophil × platelet/lymphocyte
NLR: Neutrophil/lymphocyte ratio
PLR: Platelet/lymphocyte ratio
IgG: Immunoglobulin G
DOS: Docetaxel oxaliplatin and S-1
XELOX: Capecitabine and oxaliplatin
PGC: Porous graphic carbon
TFA: Trifluoroacetic acid
ACN: Acetonitrile
UPLC: Ultra Performance Liquid Chromatography
2-AB: 2-Aminobenzamide
ROC: Receiver operating characteristic curve
AUC: Under area of ROC
ADCC: Activate antibody-dependent cellular cytotoxicity
CDC: Complement-dependent cytotoxicity
H: Mannose
N: *N*-acetylglucosamine
F: Fucose
S: *N*-acetylneuraminic acid
