# Supplementary material for: Prediction of neoadjuvant chemotherapeutic efficacy in patients with locally advanced gastric cancer by serum IgG glycomics profiling
Source: Clin Proteomics. 2020 Feb 6;17:4. doi: 10.1186/s12014-020-9267-8 (PMC7003487; doi:10.1186/s12014-020-9267-8)
Supplement: Supplementary file 1 — Additional file 1: Table S1. Summarized glycan traits based on the glycan structure. Table S2. IgG glycome composition in gastric cancer patients with response to NACT before and after treatment. Table S3. IgG glycome composition in gastric cancer patients without response to NACT before and after treatment. [file 12014_2020_9267_MOESM1_ESM.docx]

# Additional Table

**Table S1**

Summarized glycan traits based on the glycan structure

| **Trait** | **Description** | **Formula** |
| --- | --- | --- |
| GPN | The proportion of total neutral glycans | GP1+GP2+GP3+GP4+GP5+GP6+GP7+GP8+GP9+GP10+GP11+GP12+GP13+GP14+GP15 |
| S1 total | The proportion of sialylated structures in total IgG glycans | GP16+GP17+GP18+GP19 |
| S2 total | The proportion of monosialylated structures in total IgG glycans | GP21+GP22+GP23+GP24 |
| S total | The proportion of disialylatied structures in total IgG glycans | GP16+GP17+GP18+GP19+GP21+GP22+GP23+GP24 |
| G0 total | The proportion of agalactosylated structures in total IgG glycans | GP1+ GP2+ GP3+ GP4+ GP6 |
| G1 total | The proportion of monogalactosylated structures in total IgG glycans | GP7+ GP8+ GP9+ GP10+ GP11 |
| G2 total | The proportion of digalactosylated structures in total IgG glycans | GP12+ GP13+ GP14+ GP15 |
| F total | The proportion of fucosylated structures in total IgG glycans | GP1+ GP4+ GP6+ GP8+ GP9+ GP10+ GP11+ GP14+ GP15+ GP16+ GP18+ GP19+ GP23+ GP24 |
| F neutral | The proportion of fucosylated structures in total neutral glycans | (GP1+ GP4+ GP6+ GP8+ GP9+ GP10+ GP11+ GP14+ GP15)/GPN*100 |
| F sialo | The proportion of fucosylated structures in total sialylated glycans | (GP16+ GP18+ GP19+ GP23+ GP24)/GPS*100 |
| B total | The proportion of structures with bisecting GlcNAc in total IgG glycans | GP3+ GP6+ GP10+ GP11+ GP13+ GP15+ GP19+ GP22+ GP24 |
| B neutral | The proportion of fucosylated structures with bisecting GlcNAc in total neutral glycans | (GP3+ GP6+ GP10+ GP11+ GP13)/GPN*100 |
| B sialo | The proportion of fucosylated structures with bisecting GlcNAc in total sialylated glycans | (GP19+ GP22+ GP24)/GPS*100 |
| Gal-ratio | the ratio of agalactosylated structures to galactosylated structures of fucosylated glycans | FG0/(FG1+FG2*2) |
| FG0 | The proportion of agalactosylated structures in total fucosylated glycans | GP4/F total |
| FG1 | The proportion of monogalactosylated structures in total fucosylated glycans | (GP8+GP9)/F total |
| FG2 | The proportion of digalactosylated structures in total fucosylated glycans | GP14/F total |

**Table S2**

IgG glycome composition in gastric cancer patients with response to NACT before and after treatment.

| **IgG glycome** | **Traits (Composition)** | **Mean (before NACT)** | **Mean (after NACT)** | **P value** |
| --- | --- | --- | --- | --- |
| Directly detected glycans | GP1 (H3N3F1) | 0.18 | 0.14 | 0.10 |
|  | GP2 (H3N4) | 0.78 | 0.80 | 0.87 |
|  | GP3 (H3N5) | 0.00 | 0.00 | ns |
|  | GP4 (H3N4F1) | 27.11 | 25.67 | 0.45 |
|  | GP5 (H5N2) | 0.13 | 0.14 | 0.69 |
|  | GP6 (H3N5F1) | 7.54 | 7.78 | 0.66 |
|  | GP7 (H4N4) | 0.33 | 0.35 | 0.65 |
|  | GP8 (H4N4F1(6)) | 19.70 | 19.93 | 0.76 |
|  | GP9 (H4N4F1(3)) | 9.92 | 9.92 | 1.00 |
|  | GP10 (H4N5F1(6)) | 6.12 | 6.80 | 0.13 |
|  | GP11 (H4N5F1(3)) | 0.71 | 0.74 | 0.56 |
|  | GP12 (H5N4) | 0.82 | 0.84 | 0.91 |
|  | GP13 (H5N5) | 0.00 | 0.00 | ns |
|  | GP14 (H5N4F1) | 13.28 | 13.00 | 0.83 |
|  | GP15 (H5N5F1) | 1.50 | 1.62 | 0.44 |
|  | GP16 (H4N4F1S1(3)) | 2.22 | 2.27 | 0.74 |
|  | GP17 (H5N4S1) | 0.51 | 0.57 | 0.27 |
|  | GP18 (H5N4F1S1) | 6.02 | 6.14 | 0.86 |
|  | GP19 (H5N5F1S1) | 0.74 | 0.85 | 0.15 |
|  | GP20 (H5N4F2S1) | 0.00 | 0.00 | ns |
|  | GP21 (H5N4S2) | 0.52 | 0.52 | 1.00 |
|  | GP22 (H5N5S2) | 0.00 | 0.00 | ns |
|  | GP23 (H5N4F1S2) | 0.82 | 0.79 | 0.78 |
|  | GP24 (H5N5F1S2) | 1.03 | 1.14 | 0.60 |
| Main summarized traits | GPN | 88.12 | 87.73 | 0.65 |
|  | S1 total | 9.50 | 9.82 | 0.65 |
|  | S2 total | 2.38 | 2.44 | 0.85 |
|  | S total | 11.88 | 12.27 | 0.65 |
|  | G0 total | 35.62 | 34.40 | 0.61 |
|  | G1 total | 36.77 | 37.74 | 0.22 |
|  | G2 total | 15.60 | 15.46 | 0.92 |
|  | F total | 94.95 | 94.72 | 0.33 |
|  | F neutral | 97.65 | 97.58 | 0.76 |
|  | F sialo | 91.11 | 91.04 | 0.91 |
|  | B total | 17.64 | 18.93 | 0.12 |
|  | B neutral | 17.96 | 19.29 | 0.13 |
|  | B sialo | 14.77 | 15.91 | 0.50 |
|  | Gal-ratio | 0.52 | 0.49 | 0.62 |
|  | FG0 | 0.29 | 0.27 | 0.47 |
|  | FG1 | 0.31 | 0.32 | 0.69 |
|  | FG2 | 0.14 | 0.14 | 0.85 |

**Table S3**

IgG glycome composition in gastric cancer patients without response to NACT before and after treatment.

| **IgG glycome** | **Traits (Composition)** | **Mean (before NACT)** | **Mean (after NACT)** | **P value** |
| --- | --- | --- | --- | --- |
| Directly detected glycans | GP1 (H3N3F1) | 0.15 | 0.13 | 0.51 |
|  | GP2 (H3N4) | 0.58 | 0.63 | 0.66 |
|  | GP3 (H3N5) | 0.00 | 0.00 | ns |
|  | GP4 (H3N4F1) | 22.40 | 21.87 | 0.70 |
|  | GP5 (H5N2) | 0.14 | 0.14 | 0.73 |
|  | GP6 (H3N5F1) | 6.86 | 7.01 | 0.68 |
|  | GP7 (H4N4) | 0.36 | 0.34 | 0.73 |
|  | GP8 (H4N4F1(6)) | 20.20 | 21.24 | 0.31 |
|  | GP9 (H4N4F1(3)) | 9.89 | 9.37 | 0.43 |
|  | GP10 (H4N5F1(6)) | 6.80 | 7.19 | 0.26 |
|  | GP11 (H4N5F1(3)) | 0.70 | 0.75 | 0.35 |
|  | GP12 (H5N4) | 0.97 | 0.89 | 0.73 |
|  | GP13 (H5N5) | 0.00 | 0.00 | ns |
|  | GP14 (H5N4F1) | 15.69 | 15.20 | 0.58 |
|  | GP15 (H5N5F1) | 1.90 | 1.89 | 0.91 |
|  | GP16 (H4N4F1S1(3)) | 2.28 | 2.37 | 0.57 |
|  | GP17 (H5N4S1) | 0.62 | 0.59 | 0.78 |
|  | GP18 (H5N4F1S1) | 7.13 | 7.11 | 0.96 |
|  | GP19 (H5N5F1S1) | 0.80 | 0.86 | 0.24 |
|  | GP20 (H5N4F2S1) | 0.00 | 0.00 | ns |
|  | GP21 (H5N4S2) | 0.54 | 0.49 | 0.34 |
|  | GP22 (H5N5S2) | 0.00 | 0.00 | ns |
|  | GP23 (H5N4F1S2) | 0.86 | 0.79 | 0.42 |
|  | GP24 (H5N5F1S2) | 1.13 | 1.15 | 0.88 |
| Main summarized traits | GPN | 86.65 | 86.65 | 1.00 |
|  | S1 total | 10.82 | 10.92 | 0.90 |
|  | S2 total | 2.53 | 2.43 | 0.72 |
|  | S total | 13.35 | 13.35 | 1.00 |
|  | G0 total | 29.99 | 29.65 | 0.82 |
|  | G1 total | 37.96 | 38.88 | 0.26 |
|  | G2 total | 18.56 | 17.98 | 0.61 |
|  | F total | 94.73 | 94.79 | 0.90 |
|  | F neutral | 97.62 | 97.69 | 0.86 |
|  | F sialo | 91.46 | 91.96 | 0.45 |
|  | B total | 18.19 | 18.85 | 0.41 |
|  | B neutral | 18.80 | 19.45 | 0.45 |
|  | B sialo | 14.47 | 15.22 | 0.59 |
|  | Gal-ratio | 0.37 | 0.37 | 0.98 |
|  | FG0 | 0.24 | 0.23 | 0.70 |
|  | FG1 | 0.32 | 0.32 | 0.53 |
|  | FG2 | 0.17 | 0.16 | 0.59 |
